# Patient and healthcare provider reported barriers and enablers to virtual or remote-only follow-up models for cardiovascular implantable electronic devices: protocol for a qualitative framework synthesis

**DOI:** 10.1186/s13643-020-01410-w

**Published:** 2020-06-24

**Authors:** Shannon E. Kelly, Tammy J. Clifford, Becky Skidmore, David Birnie, Ratika Parkash, George A. Wells

**Affiliations:** 1 School of Epidemiology and Public Health, University of Ottawa, Ottawa, Ontario Canada; 2 University of Ottawa Heart Institute, Ottawa, Ontario Canada; 3 Independent Information Specialist, Ottawa, Ontario Canada; 4 Division of Cardiology, Dalhousie University, Halifax, Nova Scotia Canada

**Keywords:** Remote monitoring, Virtual care, Model of care, Distance factors, Cardiovascular implantable electronic device, ICD, Pacemaker, CRT, Patient satisfaction, Healthcare provider, Barriers

## Abstract

**Background:**

Virtual care models are used to follow-up patients with cardiovascular implantable electronic devices (CIED), including pacemakers, implantable cardioverter defibrillators, and cardiac resynchronization therapy. There is increasing interest in the expansion of virtual, or even remote-only, CIED care models to alleviate resource and economic burden to both patients and specialty device clinics and to maintain or improve equity and access to high-quality cardiovascular care. This qualitative framework synthesis aims to identify barriers and enablers to virtual care models from both the perspective of the patient and device clinics. How setting, context, equity factors or other aspects influence these factors, or satisfaction with care, will also be investigated.

**Methods:**

We will perform a systematic literature search in MEDLINE, Embase, PsycINFO, CINAHL, Proquest Dissertations & Theses, other EBM Reviews, and trial registry databases. Screening will be completed by two independent review authors. Original research articles having a qualitative component (i.e., qualitative, mixed-, or multi-method) are eligible. Study populations of interest are (a) individuals with a CIED or (b) healthcare providers involved in any aspect of virtual or remote follow-up of patients with CIEDs. Eligibility will be restricted to studies published after January 1, 2000 in English or French. Data will be captured using standardized templates based on the domains and constructs of the Theoretical Domains Framework and the Warwick Patient Experiences Framework. The Joanna Briggs Institute Critical Appraisal Checklist for Qualitative Research will be applied to all included studies. The GRADE-CERQual approach will be applied to assess and summarize confidence in key findings. Reporting will follow the enhancing transparency in reporting the synthesis of qualitative research (ENTREQ) statement. Detailed descriptive results will be presented, and summary of qualitative findings tables will be produced.

**Discussion:**

While a number of trials have captured the clinical effectiveness and safety of virtual follow-up for CIEDs, there has been less attention given to factors affecting use and implementation of remote care by patients and healthcare providers or satisfaction with care. Results from this qualitative framework synthesis will provide important lived experience data from both patients and healthcare providers which will be essential to incorporate in clinical guidelines.

**Systematic review registration:**

PROSPERO CRD42020160533.

## Background

Patients with cardiovascular electronic implantable devices (CIEDs), including pacemakers, implantable cardioverter defibrillators (ICDs), and cardiac resynchronization therapy (CRT), are managed by specialty device outpatient clinics who provide lifelong follow-up. Patients are typically seen in clinic for follow-up appointments every three to 12 months, provisional on the device implanted, personal health status, physician preference, and device recall or alert status. Busy device clinics struggle to balance available resources with the consistently increasing number of patients who require CIED follow-up appointments [1, 2]. Virtual outpatient visits (also referred to as remote interrogation or monitoring) offer an alternative model of care to supplement, delay or alternate, but not replace, traditional in-clinic visits. Virtual follow-up offers patients the convenience of staying home while obtaining quality care for themselves and their CIED. This model of care permits clinics to follow patients with normal device function and adequate battery longevity routinely or to monitor patients under device recalls, alerts or, who are close to requiring replacement of the device, leads or battery more closely [2]. Guidelines in both Canada and the USA now recommend an alternating virtual/in-clinic model for CIEDs as standard of care [2, 3].

There is increasing interest in the expansion of virtual CIED care models to alleviate resource and economic burden to both patients and specialty device clinics and to improve equity and access to high-quality cardiovascular care. Clinical investigations are ongoing to examine the impact to patients and device clinics if routine in-clinic follow-up visits is jettisoned in favor of entirely virtual follow-up and surveillance [4]. Despite advancements in information technology and telecommunications that have facilitated expansion of virtual follow-up and care models for CIEDs, recent national survey data from Canada indicates that there is an unmet need in delivery of timely, uniform and efficient care for these patients [5]. Current data shows that CIED follow-up care is heterogeneous, and there are inconsistent approaches to remote monitoring used across jurisdictions. Although many clinics use virtual follow-up at least partially, implementation is disparate and variable across and within clinics. Patients generally perceive remote follow-up to be safe, effective, and efficient, yet not all device clinics offer virtual follow-up visits to all patients, and uptake has been suboptimal in patients offered the service [5].

Lack of a unified approach to in-clinic or virtual follow-up after CIED implant is at least partially attributable to deficiencies in current clinical guidelines that recommend use of virtual follow-up but without process- or context-specific direction or details on implementation. It is essential to understand how patient, clinic, or system factors influence current use and uptake of virtual models of care for patients with CIEDs to support and inform the development of new guideline-supported and promote more unified clinical approaches. The implementation and use of virtual follow-up or care may be influenced by many factors. A theoretical examination of all factors that impede and enhance the implementation and use of virtual follow-up or care is necessary in order to develop strategies to improve the consistency of care and optimize patient uptake. This study will use a framework-based qualitative synthesis approach to understand patients’ and healthcare provider barriers and facilitators to virtual follow-up or care for CIEDs, including remote-only models. Additionally, satisfaction, barriers, and facilitators will be examined and compared according to key patient, device and clinic characteristics, and by country.

## Methods

The protocol for this qualitative synthesis was written a priori and registered in the International Prospective Register of Systematic Reviews (PROSPERO, CRD42020160533). This protocol follows guidance from the Preferred Reporting Items for Systematic Review and Meta-Analysis-Protocols (PRISMA-P) statement (Additional file 1) [6]. Research questions will be addressed through a systematic, framework-based qualitative synthesis approach informed by methodological guidance for systematic reviews of complex health interventions [7–14].

Thematic synthesis is one of a number of approaches recommended by the Cochrane Qualitative Review Methods Group [11]. This approach is particularly useful when expected evidence will likely contribute thin descriptions and is likely to be predominantly descriptive (versus a highly theorized or conceptual evidence base) [15]. In the framework approach, our thematic synthesis will be guided by two theoretical frameworks selected a priori. Instead of developing a new framework after reading included studies, this review will use the Warwick Patient Experiences Framework (WaPEF) [16] and the Theoretical Domains Framework (TDF) [17, 18] for themes or categories. Both patient and device clinic (or healthcare provider) experiences with virtual follow-up or care for CIEDs will be synthesized using the TDF and the Warwick Frameworks. To understand patient experience specific to local setting or context, PROGRESS+ equity factors will be explored [19, 20]. Patients and healthcare providers who have experience and continue to use virtual follow-up will be compared to those who have used yet chose not to continue.

### Theoretical Domains Framework

The TDF is a comprehensive theoretical framework developed to facilitate the identification of factors affecting the behaviour of health professionals related to the implementation. Michie et al. proposed the TDF following a synthesis of 30+ theories of behaviour and behaviour change, and a rigorous consensus and validation process [18]. The original TDF synthesized a large number of individual behaviour change constructs into 12 distinct domains, with the goal of providing a “theoretical lens” for users to view the various environmental, cognitive, social, and affective influences on behaviour. The TDF was later put through an extensive validation process, and the individual domains and constructs were reconfigured into 14 domains by Cane et al. [17]. The TDF was selected for this review as it enables a fulsome and structured investigation into facilitators and barriers for both patient and healthcare providers, offers strength over a single model or theory approach, and provides comprehensive coverage of the possible influences on behaviour that effect the diffusion of evidence into practice [21]. It can also be applied to patient behaviours as well as healthcare providers [22]. The TDF also provides a means to progress from theories of behaviour change towards the techniques of behaviour change which will be used in future research phases to inform the design of interventions to address implementation issues and inform process evaluations. Results may also provide insight or lead to greater understanding of the processes that underlie existing non-theoretically based interventions.

### Warwick Patient Experiences Framework

The WaPEF was developed by Staniszewska et al. as a way to include patient-based evidence in guidelines alongside standard clinical and economic outcomes [16]. Important consideration so that patient experiences and perspectives can be included in eventual guidelines. It is used to collect and summarize qualitative data using a framework of generic dimensions of patient experiences and to provide an evidence base for each theme and sub-theme by linking each using comprehensive evidence tables. In this review, the WaPEF will be used to assemble and structure patient experience with virtual follow-up or care for patients with CIEDs. This will also a structured examination of patient satisfaction and any model characteristics that contribute to a positive or negative lived experiences with CIED follow-up.

### Research questions

The qualitative synthesis will address the following research questions:

1. From the patient perspective, what are the barriers and facilitators related to patient use and uptake of virtual follow-up and care models for patients with CIEDs?
2. From the healthcare provider perspective, what are the barriers and facilitators related to virtual follow-up or care program delivery or implementation for patients with CIEDs?
3. What are the barriers and facilitators to remote-only models of care based on the experience or perceptions of patients or healthcare providers?
4. How do setting, geography, context, equity or other factors influence the barriers, facilitators, or satisfaction with care?

These research questions will orient data selection, collection, and analysis toward patients’ and clinical experiences and reflect a need to identify important contextual and equity influences.

### Literature search strategy

An information specialist will design and perform the literature search following peer-review of the electronic search strategy by a second, independent information specialist using the Peer Review of Electronic Search Strategies (PRESS) Guideline Statement (29). The complete proposed search strategy is presented in Additional file 2.

Information will be identified by searching the following bibliographic databases: Using the OVID platform, Ovid MEDLINE®, including Epub Ahead of Print and In-Process & Other Non-Indexed Citations, Embase Classic + Embase, PsycINFO, and the following EBM Reviews databases: Cochrane Central Register of Controlled Trials, Cochrane Database of Systematic Reviews, Database of Abstracts of Reviews of Effects, Health Technology Assessment, and the NHS Economic Evaluation Database. We will also search CINAHL (EBSCO platform) and Proquest Dissertations & Theses Global.

An initial search strategy was designed and piloted between December 29, 2018, and January 6, 2019. The proposed strategy was run on June 26, 2019 to test for volume. Following discussion, the search was revised to include additional vocabulary and thereby increase sensitivity. The searches will be conducted in two parts. The main search will apply research design filters, and the second supplemental search will utilize an extensive qualitative filter. Qualitative and case studies will be the focus of the qualitative synthesis. Trials are included in the search as they may contain embedded qualitative studies and will be identified through a related quantitative systematic review of patient outcomes currently in-progress (CRD42020145210). We will use a combination of controlled vocabulary (e.g., “Remote Consultation”, “Defibrillators, Implantable”, “Cardiac Electrophysiology”) and keywords (e.g., telemonitor, pacemaker, CIED) for the concepts in all searches. We will remove animal-only citations and news items where possible from the results. Searches will be limited by date to records available after January 1, 2000 (to coincide with the first regulatory approvals of wireless, remote monitoring systems), and not limited in any other way (e.g., by language or publication status).

### Literature selection criteria

Eligible studies will be primary English- or French-language reports of original research articles having a qualitative component (i.e., qualitative, mixed-, or multi-method studies). Studies of interest will focus on eliciting ambulatory patients’ or healthcare providers’ perceptions, attitudes, experiences, viewpoints, expectations, or understanding of CIED virtual follow-up or care. Studies may also focus specifically on elucidation of factors that influence patients’ decision to initiate, continue or cease virtual follow-up or care, or provider ability to initiate, deliver, or implement quality CIED care using remote approaches, or to change follow-up approach (e.g., from a blended clinic/virtual combination to a virtual only). Factors influencing patients’ satisfaction with care are also eligible. Table 1 details the eligibility criteria to be applied using the Perspective, Setting, Phenomenon of interest, Environment, Comparison, Timing, and Findings (PerSPECTiF) framework [8].

**Table 1:** Eligibility criteria for the research questions using the PerSPECTiF framework for (a) patients or their caregivers, (b) device clinics

Per perspective	S setting	P phenomenon of interest	E environment	C comparison	Ti timing	F findings
(a) Individuals with a CIED (pacemaker, ICD, (CRT)^a.^ (b) Clinical caregiver involved in any aspect of virtual follow-up or care of patients with CIEDs.	Outpatient/ambulatory care. In any setting (urban, rural, remote) or context.	Virtual follow-up and/or virtual care^b^.	In any environment or within an environment of prioritized health equity.	Any or none.	Any time following CIED implant (short or long-term). Any timing of intervention delivery.	Barriers, facilitators, and satisfaction with care as identified through perceptions, attitudes, experiences, viewpoints, expectations, understandings of patients or healthcare providers when using or implementing virtual follow-up or care (using any service model or approach).
Study design						
Original research articles having a qualitative component (i.e., qualitative, mixed-, or multi-method studies).						
Time frame						
January 1, 2000 to present.						
Language						
English or French.						

*CIED* cardiovascular implantable electronic device, *ICD* implantable cardioverter defibrillator, *CRT* cardiac resynchronization therapy

^a^Device may be de novo, existing, or in a patient undergoing a pulse generator change that now has virtual follow-up or care capabilities, or their informal caregivers

^b^Includes the broad context in which virtual follow-up or care is used (e.g., setting, resource allocation considerations, health, and human resources issues); how it fits in the process of patient care; experiences, expectations, and perceptions of virtual follow-up or care

All studies will be included regardless of comparability of health care systems; transferability or generalizability (i.e., external validity) will be considered during data extraction, critical appraisal, and analysis.

The following definitions for virtual follow-up and care will be used:

1. *Virtual follow-up* may also be referred to as remote monitoring. In this study, we broadly include the collection of device or patient data and/or diagnostics via passive remote device interrogations and the automated transmission of active pre-specified alerts related to device functionality and clinical events. This involves a one-way transmission of data from the patient in their outpatient setting to a receptor device or specialty clinic. Here, the patient alternates virtual follow-up from home with in-person device clinic visits (6-month intervals for ICDs/CRT, 12-month intervals for pacemakers). This approach can be utilized for any CIED (pacemakers, ICDs, or CRTs) which have the capability. This may include remote-only models of care. Virtual follow-up is most-often suited for patients with stable device function and adequate battery longevity after at least one in-person post-surgical follow-up visit.
2. *Virtual care* may also be referred to as remote patient management and involves therapeutic intervention on the patient’s implanted CIED from a distance using available technology (e.g., remotely re-programming device thresholds and automated recalibration of device settings using machine-learning algorithms). This involves two-way interaction and is informed by virtual follow-up through transmission of data from the patient in their outpatient (or non-device clinic) setting to a receptor device clinic and then related care or action involving some kind of therapeutic adjustment from a physician at the receptor device clinic back to the patient’s device. Any virtual therapeutic intervention for CIED patients is considered in-scope for this review.

Both approaches may also be accompanied by concurrent interventions aimed at patient self-efficacy and empowerment (e.g., providing a patient with their own data, software, or mobile phone applications).

Findings are representative of outcomes for the phenomena of interest and while prioritization of barriers and facilitators of interest (or factors influencing satisfaction with care) is not appropriate in advance, reporting of results will highlight factors which may be modifiable, contribute most to desired changes in patient or provider behaviours, and/or be more likely to be measurable in some way in future research.

### Literature screening and selection

One reviewer will screen the titles and abstracts of all citations retrieved against the eligibility criteria (see Table 1). A second independent reviewer will screen all titles and abstracts excluded by the first reviewer, and a citation will not be excluded during screening at the title and abstract stage unless both reviewers agree. All citations deemed potentially relevant (or unclear) at the title, and abstract stage will be retrieved as full-text articles for a second level of screening by two independent review authors. Discrepancies will be resolved through discussion or consultation with a third reviewer if necessary. We will use standardized forms for article screening and selection set up through online systematic review management software (e.g., DistillerSR). The full literature screening and selection process will be documented and presented in a PRISMA flow diagram [23].

### Data-extraction and synthesis approach

All data extraction will be completed by one reviewer and checked for completeness and accuracy by a second independent reviewer. Extraction of data for all included studies will be done in Microsoft Excel using forms customized for this review and standardized in advance. Where there are multiple reports for a single study, we will extract data from all reports into one form and document the related citations. A data extraction form will be developed to capture the domains and constructs of the TDF and the WaPEF, along with a study design and participant characteristics. The frameworks allow for thematic and theory-informed extraction of key data from all included studies. A single review author will independently review the key findings and conclusions of the eligible studies and extract all barriers, facilitators (SK), or factors related to patient satisfaction. These data will be checked by a second independent review author for consistency and completeness. Extracted data will consist of verbatim text from the original publication and include participant quotations and/or associated interpretive descriptions from the study authors alongside the summaries of results as appropriate. All barrier, enabler, and satisfaction data will be collected regardless of duplication across studies.

One review author will independently code relevant text directly into the a priori domains (and sub-domains or themes) of the WaPEF and TDF domains and code any additional data in an “other” domain if not covered by the existing frameworks. The WaPEF will be used to code patient satisfaction data, and the TDF will be used for both patient and healthcare provider barriers and facilitators. A second independent review author will check the coding completed by the first author in its entirety, and any disagreements will be resolved through discussion, with input from a third review author if necessary, for consensus.

Additional information will be extracted from all included studies, including bibliographic information details pertinent to study characteristics (e.g., first author, publication year, and country), research methodology (study design, aim or objectives, methods of data collection, source of barrier/facilitator data extraction), and population (e.g., type of participants, number of patients/healthcare providers/clinics, age, sex/gender, and type of device).

The thematic synthesis will be developed by arranging individual study data by domain into tables to both explore sub-domains within each framework and to provide a means to develop refined summaries of evidence across studies. Using the charts, the range and nature of the data for virtual follow-up and care models will be explored separately for patients and for healthcare providers. Data within and across domains (including subdomains) will be used to identify barriers and facilitators common across or unique to patients and health providers, as well as apparent and potentially unexplored gaps in the literature. Local or regional context, type of CIED, sex, age, setting (urban, rural, remote), and PROGRESS-PLUS equity factors [19, 24] will be explored within the extracted data across and within domains. Identified barriers will be classified as modifiable or non-modifiable and will provide a platform to explore potentially relevant behavioural change techniques aimed at modifiable barriers with the goal of improving quality of care, uptake, and implementation in future research.

### Critical appraisal of individual studies

A critical appraisal of included studies will be conducted by one reviewer and checked by a second independent reviewer. All disagreements will be resolved through discussion or consultation with a third reviewer as needed. Results from the appraisal will be summarized narratively to highlight strengths and limitations within and across studies. Tables or figures will be used to present and/or graphically summarize results.

The Joanna Briggs Institute (JBI) Critical Appraisal Checklist for Qualitative Research will be used to appraise all included qualitative research studies. Judgements on the ten checklist questions pertaining to the methodological quality of the study using the answers provided, including “yes,” “no,” “unclear,” and “no information.” In this checklist, “yes’ answers indicate stronger study quality. In addition to the JBI checklist, reviewers will globally consider major strengths and limitations of studies included in terms of credibility, transferability, dependability, and confirmability and will document results by study. Studies that meet the eligibility criteria will be included regardless of study quality.

There is currently no consensus on the role of quality criteria and how they should be applied, and there is ongoing debate about how qualitative study quality should be assessed for the purposes of systematic reviews [25]. As such, the quality assessment will be used when judging the relative contribution of each study to the development of explanations and relationships, and reviewers will use critical appraisal results to broadly consider both the internal validity and reliability of the research. Following an approach used by CADTH, studies deemed to be lacking in one or both of these areas may be coded last so they do not lead the analysis. Likewise, the transferability of the research findings will be considered during analysis and reporting.

### Certainty of evidence

The GRADE-CERQual (“Confidence in the Evidence from Reviews of Qualitative research”) approach will be applied to assess and summarize confidence in key findings, including the methodological limitations of the individual qualitative studies contributing to a review finding, the coherence of the review finding, the adequacy of data supporting a review finding, and the relevance to the review question of the individual studies contributing to a review finding [9]. A fifth component, dissemination (or publication) bias will also be explored [26]. This will provide overall confidence in each of the key findings. Key research findings are defined as the set of themes, concepts, or perceptions or experiences identified as having the most relevance to the research question.

A single reviewer will independently assess certainty of the evidence using the GRADE-CERQual approach. A second independent review author will verify results. Disagreements will be resolved through discussion and documented to facilitate reviewer conclusions pertaining to confidence and transparency of study findings. Additional GRADE guidance currently under development in an ongoing research project will be used to more specifically contextualize the assessment for considerations relevant to complex health interventions [10]. This approach facilitates incorporation of evidence from frameworks into the GRADE assessment and outlines additional considerations relating to context, setting, and other factors that assessors can use in rating the certainty of evidence. Results will be presented in GRADE-CERQual summary of qualitative findings tables.

## Discussion

While a number of primary research studies have examined the clinical effectiveness and safety of virtual follow-up for CIEDs, there has been less attention given to factors affecting use and implementation of remote care by patients and healthcare providers, and, to our knowledge, this literature has never been synthesised. This review will be a comprehensive synthesis of the qualitative literature on virtual follow-up or care for CIEDs and will be the first to apply a theory-based framework assessment to this body of evidence. Results will inform the development and application to theory-based behavioural change techniques that aimed at improving consistency and quality of care. Results will be informative to a variety of knowledge users, including patients, informal caregivers, health providers, and policy makers. Results will complement an associated quantitative systematic review of context and setting-specific effectiveness and safety to inform future work and research aiming to generally expand access, uptake, use and spread of virtual follow-up, and care models for CIED patients. Globally, results may also inform advances in post-implant care in high-, middle-, or low-income countries. Ideally, new practice guidelines for management of this population will be developed that adequately consider all evidence relevant to the delivery of context and equity-sensitive patient-centred arrhythmia management strategies for virtual follow-up and care.

There are potential limitations associated with relying on data reported and interpreted at the individual study level. Possible limitations include a potential for biased reporting in the original studies if selective findings are presented to fit any stated research question(s). In this case, results or data that are relevant to this review may not have been reported completely. The review is also reliant on the descriptions of results in the eligible studies, and the granularity of results may be inadequate which is also a potential limitation.

## Reporting

Reporting for this study will follow the enhancing transparency in reporting the synthesis of qualitative research (ENTREQ) statement [38]. All deviations from the original review protocol will be documented and reported in full in the final review publication.

## Supplementary information

**Additional file 1.** PRISMA-P 2015 Checklist.

**Additional file 2.** Proposed search strategy.

## Data Availability

Not applicable.

## Abbreviations

CERQual: Confidence in the Evidence from Reviews of Qualitative Research
CIED: Cardiovascular implantable electronic device
CRT: Cardiac resynchronization therapy
ENTREQ: Enhancing Transparency in Reporting the Synthesis of Qualitative Research
GRADE: Grading of Recommendations, Assessment, Development and Evaluations
ICD: Implantable cardioverter defibrillator
JBI: Joanna Briggs Institute
PRISMA: Preferred Reporting Items for Systematic Reviews and Meta-Analyses
PROGRESS-Plus: Place of residence, race/ethnicity, occupation, gender, religion/culture, education, socio-economic status, social capital/networks, plus other important factors which impact on health equity
RCT: Randomized controlled trial
TDF: Theoretical Domains Framework (TDF)
WaPEF: Warwick Patient Experiences Frameworks
