# Supplementary material for: Patient and healthcare provider reported barriers and enablers to virtual or remote-only follow-up models for cardiovascular implantable electronic devices: protocol for a qualitative framework synthesis
Source: Syst Rev. 2020 Jun 24;9:151. doi: 10.1186/s13643-020-01410-w (PMC7315548; doi:10.1186/s13643-020-01410-w)
Supplement: Supplementary file 2 — Additional file 2. Proposed search strategy. [file 13643_2020_1410_MOESM2_ESM.docx]

| Additional file 2. Proposed search strategy | |
| --- | --- |
| RESEARCH DESIGN SEARCHES (Ovid Multifile)  Database: Embase Classic+Embase <1947 to 2019 June 25>, Ovid MEDLINE(R) ALL <1946 to June 25, 2019>, PsycINFO <1806 to June Week 3 2019>, EBM Reviews - Cochrane Central Register of Controlled Trials <May 2019>, EBM Reviews - Cochrane Database of Systematic Reviews <2005 to June 26, 2019> |  |
| 1 Monitoring, Ambulatory/ (18986)  2 Electrocardiography, Ambulatory/ (11916)  3 Telemedicine/ (47095)  4 Remote Consultation/ (13263)  5 Remote Sensing Technology/ (9944)  6 Telemetry/ (28300)  7 (remote* adj3 (manag* or monitor*)).tw,kf. (10240)  8 ((outpatient* or out-patient* or ambulator* or home? or homebased or home-based) adj3 (manag* or monitor*)).tw,kf. (96792)  9 ((dynamic* or Holter) adj3 (manag* or monitor*)).tw,kf. (30600)  10 ((internet* or app or apps or smarthome* or smart-home* or smartphone* or smart phone* or mobile-based or e-mail* or email* or electronic mail* or emedicine or e-medicine or technolog* or computer* or digital* or webbased or web-based or webdeliver* or web-deliver* or online) adj3 (manag* or monitor*)).tw,kf. (45175)  11 (telemonitor* or tele-monitor*).tw,kf. (4780)  12 (telecare or tele-care or telehealth or tele-health or telemedicine or tele-medicine).tw,kf. (36751)  13 (ehealth or e-health).tw,kf. (12584)  14 (mobile health or mhealth or m-health).tw,kf. (12163)  15 (telemetr* or tele-metr*).tw,kf. (23569)  16 (remote sensing or remote sensor*).tw,kf. (12966)  17 or/1-16 [REMOTE MONITORING] (327016)  18 exp Cardiac Pacing, Artificial/ (66675)  19 (artificial* adj2 card* pac*).tw,kf. (412)  20 (cardiac adj (resynchroni* or re-synchroni*)).tw,kf. (22421)  21 exp Pacemaker, Artificial/ (76313)  22 (pacemak* or pace-mak*).tw,kf. (97282)  23 Defibrillators, Implantable/ (32763)  24 (implant* adj3 defibrillat*).tw,kf. (39103)  25 (implant* adj3 device*).tw,kf. (48148)  26 ((cardiac* or cardio*) adj3 implant*).tw,kf. (46508)  27 (ICD or ICDs).tw,kf. and (exp Heart/ or (cardio* or cardiac* or coronar* or heart? or defibrillat* or fibrillat* or implant*).tw,kf.) (41971)  28 (AICD or AICDs or S-ICD or S-ICDs or CIED or CIEDs).tw,kf. (6774)  29 "Atlas II".tw,kf. (16)  30 (AnalyST Accel or Belos or Lexos or Tachos).tw,kf. (23)  31 (Durata or Dynagen or Energen).tw,kf. (322)  32 Epic DR.tw,kf. (0)  33 "Ilivia 7".tw,kf. (0)  34 Incepta.tw,kf. (19)  35 InSync.tw,kf. (233)  36 (Intica or Inventra or Iperia 7).tw,kf. (2)  37 (Lumax or Lumos).tw,kf. and (exp Heart/ or (cardio* or cardiac* or coronar* or heart? or defibrillat* or fibrillat* or implant*).tw,kf.) (63)  38 ("Maximo II" or "Maximo 2").tw,kf. (8)  39 Micro Jewel.tw,kf. (10)  40 Paradym.tw,kf. (29)  41 Riata Model 7070.tw,kf. (0)  42 Teligen.tw,kf. (19)  43 Ventak.tw,kf. (178)  44 ("remote view" and Medtronic).tw,kf. (0)  45 Electrophysiology/ (178967)  46 Cardiac Electrophysiology/ (18878)  47 (electrophysiolog* or electro-physiolog*).tw,kf. (266829)  48 Monitoring, Physiologic/ and (exp Heart/ or (cardio* or cardiac* or coronar* or heart? or defibrillat* or fibrillat* or implant*).tw,kf.) (15696)  49 or/18-48 [ICDs] (659023)  50 17 and 49 [REMOTE MONITORING - ICDs] (16917)  51 limit 50 to "systematic reviews" [Limit not valid in Embase,CCTR,CDSR; records were retained] (9308)  52 systematic review.pt. (116820)  53 meta analysis.pt. (102796)  54 exp meta-analysis as topic/ (58375)  55 (meta-analy* or metanaly* or metaanaly* or met analy* or integrative research or integrative review* or integrative overview* or research integration or research overview* or collaborative review*).tw,kf. (416016)  56 (systematic review* or systematic overview* or evidence-based review* or evidence-based overview* or (evidence adj3 (review* or overview*)) or meta-review* or meta-overview* or meta-synthes* or rapid review* or "review of reviews" or technology assessment* or HTA or HTAs).tw,kf. (488701)  57 exp Technology assessment, biomedical/ (24465)  58 (cochrane or health technology assessment or evidence report).jw. (50305)  59 (network adj (MA or MAs)).tw,kf. (25)  60 (NMA or NMAs or MTC or MTCs or MAIC or MAICs).tw,kf. (16736)  61 indirect* compar*.tw,kf. (6012)  62 (indirect treatment* adj1 compar*).tw,kf. (843)  63 (mixed treatment* adj1 compar*).tw,kf. (1468)  64 (multiple treatment* adj1 compar*).tw,kf. (424)  65 (multi-treatment* adj1 compar*).tw,kf. (8)  66 simultaneous* compar*.tw,kf. (2598)  67 mixed comparison?.tw,kf. (125)  68 or/52-67 (851174)  69 50 and 68 (383)  70 51 or 69 [REVIEWS] (9401)  71 (controlled clinical trial or randomized controlled trial).pt. (1131331)  72 clinical trials as topic.sh. (220658)  73 Randomized Controlled Trials as Topic/ (228469)  74 (randomi#ed or randomi#ation? or randomly or RCT? or placebo*).tw,kf. (3289901)  75 ((singl* or doubl* or trebl* or tripl*) adj (mask* or blind* or dumm*)).tw,kf. (675180)  76 trial.ti. (787404)  77 or/71-76 (4081226)  78 50 and 77 [RCTs] (2516)  79 controlled clinical trial.pt. (184065)  80 Controlled Clinical Trial/ or Controlled Clinical Trials as Topic/ (570947)  81 (control* adj2 trial*).tw,kf. (870984)  82 Non-Randomized Controlled Trials as Topic/ (10735)  83 (nonrandom* or non-random* or quasi-random* or quasi-experiment*).tw,kf. (151292)  84 (nRCT or nRCTs or non-RCT?).tw,kf. (2577)  85 Controlled Before-After Studies/ (214554)  86 (control* adj3 ("before and after" or "before after")).tw,kf. (652139)  87 Interrupted Time Series Analysis/ (206770)  88 time series.tw,kf. (68137)  89 (pre- adj3 post-).tw,kf. (260855)  90 (pretest adj3 posttest).tw,kf. (20565)  91 Historically Controlled Study/ (224923)  92 (control* adj2 stud$3).tw,kf. (653021)  93 Control Groups/ (126085)  94 (control* adj2 group$1).tw,kf. (1430818)  95 trial.ti. (787404)  96 or/79-95 (4255205)  97 50 and 96 [NON-RCTs] (1966)  98 exp Cohort Studies/ (2490524)  99 cohort?.tw,kf. (1523516)  100 Retrospective Studies/ (1185723)  101 (longitudinal or prospective or retrospective).tw,kf. (3313689)  102 ((followup or follow-up) adj (study or studies)).tw,kf. (137922)  103 Observational study.pt. (64534)  104 (observation$2 adj (study or studies)).tw,kf. (265873)  105 ((population or population-based) adj (study or studies or analys#s)).tw,kf. (43589)  106 ((multidimensional or multi-dimensional) adj (study or studies)).tw,kf. (373)  107 Comparative Study.pt. (1996968)  108 ((comparative or comparison) adj (study or studies)).tw,kf. (286904)  109 exp Case-Control Studies/ (1173812)  110 ((case-control* or case-based or case-comparison) adj (study or studies)).tw,kf. (239419)  111 Cross-Sectional Studies/ (478952)  112 (cross-section* or crosssection*).tw,kf. (891689)  113 or/98-112 (8605399)  114 50 and 113 [OBSERVATIONAL STUDIES] (4560)  115 Case Reports.pt. (2029254)  116 (case report? or case series? or case study or case studies).tw,kf. (1330759)  117 115 or 116 (3044931)  118 50 and 117 [CASE STUDIES] (1179)  119 70 or 78 or 97 or 114 or 118 [ALL STUDY DESIGNS] (13081)  120 exp Animals/ not Humans/ (18733129)  121 119 not 120 [ANIMAL-ONLY REMOVED] (8525)  122 (news or newspaper article).pt. (212926)  123 121 not 122 [NEWS ITEMS REMOVED] (8523)  124 limit 123 to yr="2000-current" (5392)  125 124 use medall [MEDLINE RECORDS] (2191)  126 ambulatory monitoring/ (19499)  127 ambulatory electrocardiography/ (11916)  128 telemedicine/ (47095)  129 telecardiology/ (235)  130 teleconsultation/ (13818)  131 telemonitoring/ (2669)  132 telehealth/ (29526)  133 remote sensing/ (7438)  134 telemetry/ (28300)  135 (remote* adj3 (manag* or monitor*)).tw,kw. (10347)  136 ((outpatient* or out-patient* or ambulator* or home? or home-based) adj3 (manag* or monitor*)).tw,kw. (98381)  137 ((dynamic* or Holter) adj3 (manag* or monitor*)).tw,kw. (30996)  138 ((internet* or app or apps or smarthome* or smart-home* or smartphone* or smart phone* or mobile-based or e-mail* or email* or electronic mail* or emedicine or e-medicine or technolog* or computer* or digital* or webbased or web-based or webdeliver* or web-deliver* or online) adj3 (manag* or monitor*)).tw,kw. (45664)  139 (telemonitor* or tele-monitor*).tw,kw. (4982)  140 (telecare or tele-care or telehealth or tele-health or telemedicine or tele-medicine).tw,kw. (39898)  141 (ehealth or e-health).tw,kw. (13969)  142 (mobile health or mhealth or m-health).tw,kw. (13329)  143 (telemetr* or tele-metr*).tw,kw. (24210)  144 (remote sensing or remote sensor*).tw,kw. (14010)  145 or/126-144 [REMOTE MONITORING] (333631)  146 exp heart pacing/ (41342)  147 (artificial* adj2 card* pac*).tw,kw. (517)  148 (cardiac adj (resynchroni* or re-synchroni*)).tw,kw. (23188)  149 exp artificial heart pacemaker/ (49285)  150 (pacemak* or pace-mak*).tw,kw. (99085)  151 exp implantable cardioverter defibrillator/ (53186)  152 (implant* adj3 defibrillat*).tw,kw. (40891)  153 (implant* adj3 device*).tw,kw. (48521)  154 ((cardiac* or cardio*) adj3 implant*).tw,kw. (48697)  155 (ICD or ICDs).tw,kw,dv. and (exp heart/ or (cardio* or cardiac* or coronar* or heart? or defibrillat* or fibrillat* or implant*).tw,kw.) (42597)  156 (AICD or AICDs or S-ICD or S-ICDs or CIED or CIEDs).tw,kw,dv. (6902)  157 "Atlas II".tw,kw,dv. (39)  158 (AnalyST Accel or Belos or Lexos or Tachos).tw,kw,dv. (60)  159 (Durata or Dynagen or Energen).tw,kw,dv. (396)  160 Epic DR.tw,kw,dv. (11)  161 "Ilivia 7".tw,kw,dv. (0)  162 Incepta.tw,kw,dv. (31)  163 InSync.tw,kw,dv. (461)  164 (Intica or Inventra or Iperia 7).tw,kw,dv. (11)  165 (Lumax or Lumos).tw,kw,dv. and (exp heart/ or (cardio* or cardiac* or coronar* or heart? or defibrillat* or fibrillat* or implant*).tw,kw.) (177)  166 ("Maximo II" or "Maximo 2").tw,kw,dv. (42)  167 Micro Jewel.tw,kw,dv. (38)  168 Paradym.tw,kw,dv. (49)  169 Riata Model 7070.tw,kw,dv. (1)  170 Teligen.tw,kw,dv. (61)  171 Ventak.tw,kw,dv. (329)  172 ("remote view" and Medtronic).tw,kw,dv. (1)  173 electrophysiology/ (178967)  174 heart electrophysiology/ (18465)  175 (electrophysiolog* or electro-physiolog*).tw,kw. (274744)  176 physiologic monitoring/ and (exp heart/ or (cardio* or cardiac* or coronar* or heart? or defibrillat* or fibrillat* or implant*).tw,kw.) (16195)  177 or/146-176 [ICDs] (666890)  178 145 and 177 [REMOTE MONITORING - ICDs] (17096)  179 meta-analysis/ (272024)  180 "systematic review"/ (318048)  181 "meta analysis (topic)"/ (40255)  182 (meta-analy* or metanaly* or metaanaly* or met analy* or integrative research or integrative review* or integrative overview* or research integration or research overview* or collaborative review*).tw,kw. (424177)  183 (systematic review* or systematic overview* or evidence-based review* or evidence-based overview* or (evidence adj3 (review* or overview*)) or meta-review* or meta-overview* or meta-synthes* or "review of reviews" or technology assessment* or HTA or HTAs).tw,kw. (495077)  184 biomedical technology assessment/ (23351)  185 (cochrane or health technology assessment or evidence report).jw. (50305)  186 (network adj (MA or MAs)).tw,kw. (25)  187 (NMA or NMAs).tw,kw. (5073)  188 indirect* compar*.tw,kw. (6078)  189 (indirect treatment* adj1 comparison?).tw,kw. (843)  190 (mixed treatment* adj1 comparison?).tw,kw. (1479)  191 (multiple treatment* adj1 comparison?).tw,kw. (299)  192 (multi-treatment* adj1 comparison?).tw,kw. (7)  193 simultaneous* compar*.tw,kw. (2598)  194 mixed comparison?.tw,kw. (126)  195 or/179-194 (911670)  196 178 and 195 [REVIEWS] (427)  197 exp randomized controlled trial/ or controlled clinical trial/ (1317140)  198 exp "clinical trial (topic)"/ (298384)  199 (randomi#ed or randomi#ation or randomly or RCT$1 or placebo*).tw,kw. (3342971)  200 ((singl* or doubl* or trebl* or tripl*) adj (mask* or blind* or dumm*)).tw,kw. (698184)  201 trial.ti. (787404)  202 or/197-201 (4191929)  203 178 and 202 [RCTs] (2601)  204 controlled clinical trial/ (556944)  205 "controlled clinical trial (topic)"/ (10209)  206 (control* adj2 trial*).tw,kw. (1120826)  207 (nonrandom* or non-random* or quasi-random* or quasi-experiment*).tw,kw. (151593)  208 (nRCT or nRCTs or non-RCT$1).tw,kw. (2577)  209 (control* adj3 ("before and after" or "before after")).tw,kw. (652144)  210 time series analysis/ (23511)  211 time series.tw,kw. (69033)  212 pretest posttest control group design/ (398)  213 (pre- adj3 post-).tw,kw. (260894)  214 (pretest adj3 posttest).tw,kw. (21178)  215 controlled study/ (6755569)  216 (control* adj2 stud$3).tw,kw. (950145)  217 control group/ (125991)  218 (control* adj2 group?).tw,kw. (1431019)  219 trial.ti. (787404)  220 or/204-219 (9823815)  221 178 and 220 [NON-RCTs] (3402)  222 cohort analysis/ (720709)  223 cohort?.tw,kw. (1527903)  224 retrospective study/ (1547929)  225 longitudinal study/ (252108)  226 prospective study/ (1037499)  227 (longitudinal or prospective or retrospective).tw,kw. (3327656)  228 follow up/ (1456934)  229 ((followup or follow-up) adj (study or studies)).tw,kw. (139759)  230 observational study/ (234357)  231 (observation$2 adj (study or studies)).tw,kw. (267950)  232 population research/ (100434)  233 ((population or population-based) adj (study or studies or analys#s)).tw,kw. (51675)  234 ((multidimensional or multi-dimensional) adj (study or studies)).tw,kw. (374)  235 exp comparative study/ (3200711)  236 ((comparative or comparison) adj (study or studies)).tw,kw. (300379)  237 exp case control study/ (1173812)  238 ((case-control* or case-based or case-comparison) adj (study or studies)).tw,kw. (242508)  239 cross-sectional study/ (606524)  240 (cross-section* or crosssection*).tw,kw. (894508)  241 or/222-240 (10396399)  242 178 and 241 [OBSERVATIONAL STUDIES] (5703)  243 case study/ (2099054)  244 (case report? or case series? or case study or case studies).tw,kw. (1319570)  245 243 or 244 (3076984)  246 178 and 245 [case studies] (1096)  247 178 and 245 [CASE STUDIES] (1096)  248 196 or 203 or 221 or 242 or 247 [ALL STUDY DESIGNS] (9021)  249 exp animal/ or exp animal experimentation/ or exp animal model/ or exp animal experiment/ or nonhuman/ or exp vertebrate/ (51461499)  250 exp human/ or exp human experimentation/ or exp human experiment/ (39522295)  251 249 not 250 (11940898)  252 248 not 251 [ANIMAL-ONLY REMOVED] (8355)  253 limit 252 to yr="2000-current" (6543)  254 253 use emczd [EMBASE RECORDS] (3681)  255 Telemedicine/ (47095)  256 Telemetry/ (28300)  257 (remote* adj3 (manag* or monitor*)).tw. (10100)  258 ((outpatient* or out-patient* or ambulator* or home? or homebased or home-based) adj3 (manag* or monitor*)).tw. (96304)  259 ((dynamic* or Holter) adj3 (manag* or monitor*)).tw. (30548)  260 ((internet* or app or apps or smarthome* or smart-home* or smartphone* or smart phone* or mobile-based or e-mail* or email* or electronic mail* or emedicine or e-medicine or technolog* or computer* or digital* or webbased or web-based or webdeliver* or web-deliver* or online) adj3 (manag* or monitor*)).tw. (45080)  261 (telemonitor* or tele-monitor*).tw. (4688)  262 (telecare or tele-care or telehealth or tele-health or telemedicine or tele-medicine).tw. (34845)  263 (ehealth or e-health).tw. (11181)  264 (mobile health or mhealth or m-health).tw. (10437)  265 (telemetr* or tele-metr*).tw. (23134)  266 (remote sensing or remote sensor*).tw. (12253)  267 or/255-266 [REMOTE MONITORING] (291828)  268 (artificial* adj2 card* pac*).tw. (349)  269 (cardiac adj (resynchroni* or re-synchroni*)).tw. (22128)  270 Artificial Pacemakers/ (26104)  271 (pacemak* or pace-mak*).tw. (96383)  272 (implant* adj3 defibrillat*).tw. (38419)  273 (implant* adj3 device*).tw. (47920)  274 ((cardiac* or cardio*) adj3 implant*).tw. (45918)  275 (ICD or ICDs).tw. and (exp Heart/ or (cardio* or cardiac* or coronar* or heart? or defibrillat* or fibrillat* or implant*).tw.) (41491)  276 (AICD or AICDs or S-ICD or S-ICDs or CIED or CIEDs).tw. (6691)  277 "Atlas II".tw. (16)  278 (AnalyST Accel or Belos or Lexos or Tachos).tw. (23)  279 (Durata or Dynagen or Energen).tw. (318)  280 Epic DR.tw. (0)  281 "Ilivia 7".tw. (0)  282 Incepta.tw. (19)  283 InSync.tw. (233)  284 (Intica or Inventra or Iperia 7).tw. (2)  285 (Lumax or Lumos).tw. and (exp Heart/ or (cardio* or cardiac* or coronar* or heart? or defibrillat* or fibrillat* or implant*).tw.) (60)  286 ("Maximo II" or "Maximo 2").tw. (8)  287 Micro Jewel.tw. (10)  288 Paradym.tw. (29)  289 Riata Model 7070.tw. (0)  290 Teligen.tw. (19)  291 Ventak.tw. (178)  292 ("remote view" and Medtronic).tw. (0)  293 Electrophysiology/ (178967)  294 Electrocardiography/ (345757)  295 (electrophysiolog* or electro-physiolog*).tw. (260424)  296 Monitoring/ and (exp Heart/ or (cardio* or cardiac* or coronar* or heart? or defibrillat* or fibrillat* or implant*).tw.) (33923)  297 Medical Therapeutic Devices/ and (exp Heart/ or (cardio* or cardiac* or coronar* or heart? or defibrillat* or fibrillat* or implant*).tw.) (360)  298 or/268-297 [ICDs] (918371)  299 267 and 298 [REMOTE MONITORING - ICDs] (24173)  300 Meta Analysis/ (272024)  301 (meta-analy* or metanaly* or metaanaly* or met analy* or integrative research or integrative review* or integrative overview* or research integration or research overview* or collaborative review*).tw. (414743)  302 (systematic review* or systematic overview* or evidence-based review* or evidence-based overview* or (evidence adj3 (review* or overview*)) or meta-review* or meta-overview* or meta-synthes* or rapid review* or "review of reviews" or technology assessment* or HTA or HTAs).tw. (486902)  303 (network adj (MA or MAs)).tw. (25)  304 (NMA or NMAs or MTC or MTCs or MAIC or MAICs).tw. (16698)  305 indirect* compar*.tw. (5990)  306 (indirect treatment* adj1 compar*).tw. (825)  307 (mixed treatment* adj1 compar*).tw. (1411)  308 (multiple treatment* adj1 compar*).tw. (411)  309 (multi-treatment* adj1 compar*).tw. (8)  310 simultaneous* compar*.tw. (2598)  311 mixed comparison?.tw. (125)  312 or/300-311 (809012)  313 299 and 312 [REVIEWS] (337)  314 Clinical Trials/ (99968)  315 (randomi#ed or randomi#ation? or randomly or RCT? or placebo*).tw. (3287822)  316 ((singl* or doubl* or trebl* or tripl*) adj (mask* or blind* or dumm*)).tw. (675084)  317 trial.ti. (787404)  318 or/314-317 (3618610)  319 299 and 318 [RCTs] (2961)  320 (control* adj2 trial*).tw. (868488)  321 (nonrandom* or non-random* or quasi-random* or quasi-experiment*).tw. (151138)  322 (nRCT or nRCTs or non-RCT?).tw. (2577)  323 (control* adj3 ("before and after" or "before after")).tw. (652133)  324 Time Series/ (23558)  325 time series.tw. (67675)  326 (pre- adj3 post-).tw. (260764)  327 (pretest adj3 posttest).tw. (20559)  328 (control* adj2 stud$3).tw. (652027)  329 Experiment Controls/ (907)  330 (control* adj2 group$1).tw. (1430465)  331 trial.ti. (787404)  332 or/320-331 (3606965)  333 299 and 332 [NON-RCTs] (2423)  334 Cohort Analysis/ (720709)  335 cohort?.tw. (1521577)  336 Retrospective Studies/ (1185723)  337 exp Longitudinal Studies/ (402379)  338 (longitudinal or prospective or retrospective).tw. (3308730)  339 Followup Studies/ (685305)  340 ((followup or follow-up) adj (study or studies)).tw. (136557)  341 (observation$2 adj (study or studies)).tw. (265094)  342 ((population or population-based) adj (study or studies or analys#s)).tw. (43045)  343 ((multidimensional or multi-dimensional) adj (study or studies)).tw. (373)  344 ((comparative or comparison) adj (study or studies)).tw. (282128)  345 ((case-control* or case-based or case-comparison) adj (study or studies)).tw. (238502)  346 (cross-section* or crosssection*).tw. (890680)  347 or/334-346 (6693859)  348 299 and 347 [OBSERVATIONAL STUDIES] (4546)  349 Case Report/ (4520812)  350 (case report? or case series? or case study or case studies).tw. (1301141)  351 349 or 350 (5069464)  352 299 and 351 [CASE STUDIES] (2097)  353 313 or 319 or 333 or 348 or 352 [ALL STUDY DESIGNS] (9168)  354 limit 353 to yr="2000-current" (6861)  355 [354 use medall,emczd,coch,cctr,dare,cleed,clhta] (0)  356 354 not 355 [PSYCINFO RECORDS] (6861)  357 Monitoring, Ambulatory/ (18986)  358 Electrocardiography, Ambulatory/ (11916)  359 Telemedicine/ (47095)  360 Remote Consultation/ (13263)  361 Remote Sensing Technology/ (9944)  362 Telemetry/ (28300)  363 (remote* adj3 (manag* or monitor*)).ti,ab,kw. (10304)  364 ((outpatient* or out-patient* or ambulator* or home? or homebased or home-based) adj3 (manag* or monitor*)).ti,ab,kw. (97992)  365 ((dynamic* or Holter) adj3 (manag* or monitor*)).ti,ab,kw. (30896)  366 ((internet* or app or apps or smarthome* or smart-home* or smartphone* or smart phone* or mobile-based or e-mail* or email* or electronic mail* or emedicine or e-medicine or technolog* or computer* or digital* or webbased or web-based or webdeliver* or web-deliver* or online) adj3 (manag* or monitor*)).ti,ab,kw. (44276)  367 (telemonitor* or tele-monitor*).ti,ab,kw. (4941)  368 (telecare or tele-care or telehealth or tele-health or telemedicine or tele-medicine).ti,ab,kw. (39199)  369 (ehealth or e-health).ti,ab,kw. (13446)  370 (mobile health or mhealth or m-health).ti,ab,kw. (12859)  371 (telemetr* or tele-metr*).ti,ab,kw. (24134)  372 (remote sensing or remote sensor*).ti,ab,kw. (13984)  373 or/357-372 [REMOTE MONITORING] (330189)  374 exp Cardiac Pacing, Artificial/ (66675)  375 (artificial* adj2 card* pac*).ti,ab,kw. (514)  376 (cardiac adj (resynchroni* or re-synchroni*)).ti,ab,kw. (23173)  377 exp Pacemaker, Artificial/ (76313)  378 (pacemak* or pace-mak*).ti,ab,kw. (98958)  379 Defibrillators, Implantable/ (32763)  380 (implant* adj3 defibrillat*).ti,ab,kw. (40852)  381 (implant* adj3 device*).ti,ab,kw. (48417)  382 ((cardiac* or cardio*) adj3 implant*).ti,ab,kw. (48656)  383 (ICD or ICDs).ti,ab,kw. and (exp Heart/ or (cardio* or cardiac* or coronar* or heart? or defibrillat* or fibrillat* or implant*).ti,ab,kw.) (42253)  384 (AICD or AICDs or S-ICD or S-ICDs or CIED or CIEDs).ti,ab,kw. (6876)  385 "Atlas II".ti,ab,kw. (16)  386 (AnalyST Accel or Belos or Lexos or Tachos).ti,ab,kw. (23)  387 (Durata or Dynagen or Energen).ti,ab,kw. (289)  388 Epic DR.ti,ab,kw. (0)  389 "Ilivia 7".ti,ab,kw. (0)  390 Incepta.ti,ab,kw. (9)  391 InSync.ti,ab,kw. (234)  392 (Intica or Inventra or Iperia 7).ti,ab,kw. (2)  393 (Lumax or Lumos).ti,ab,kw. and (exp Heart/ or (cardio* or cardiac* or coronar* or heart? or defibrillat* or fibrillat* or implant*).ti,ab,kw.) (62)  394 ("Maximo II" or "Maximo 2").ti,ab,kw. (8)  395 Micro Jewel.ti,ab,kw. (10)  396 Paradym.ti,ab,kw. (29)  397 Riata Model 7070.ti,ab,kw. (0)  398 Teligen.ti,ab,kw. (18)  399 Ventak.ti,ab,kw. (178)  400 ("remote view" and Medtronic).ti,ab,kw. (0)  401 Electrophysiology/ (178967)  402 Cardiac Electrophysiology/ (18878)  403 (electrophysiolog* or electro-physiolog*).ti,ab,kw. (272903)  404 Monitoring, Physiologic/ and (exp Heart/ or (cardio* or cardiac* or coronar* or heart? or defibrillat* or fibrillat* or implant*).ti,ab,kw.) (15689)  405 or/374-404 [ICDs] (665627)  406 373 and 405 [REMOTE SENSING - ICDs] (17116)  407 limit 406 to yr="2000-current" (12229)  408 407 use coch [DSR RECORDS] (0)  409 407 use cctr [CENTRAL RECORDS] (978)  410 125 or 254 or 356 or 408 or 409 [ALL DATABASES] (10187)  411 limit 410 to yr="2013-current" (5677)  412 remove duplicates from 411 (4546)  413 410 not 411 (4510)  414 remove duplicates from 413 (3533)  415 412 or 414 [TOTAL UNIQUE RECORDS] (8079)  416 415 use medall (2941)  417 (20181227* or 20181228* or 20181229* or 2018123* or 2019*).dt. (652775)  418 416 and 417 [MEDLINE UNIQUE RECORDS - UPDATE PERIOD] (53)  419 415 use emczd (4380)  420 (20181228* or 20181229* or 2018123* or 2019*).dc. (1067577)  421 419 and 420 [EMBASE UNIQUE RECORDS - UPDATE PERIOD] (297)  422 415 use coch (83)  423 (20181228* or 20181229* or 2018123* or 2019*).up. (5703967)  424 422 and 423 [COCHRANE DSR UNIQUE RECORDS - UPDATE PERIOD] (28)  425 415 use cctr (635)  426 (201812* or 2019*).up. (32588533)  427 425 and 426 [CENTRAL UNIQUE RECORDS - UPDATE PERIOD] (429)  428 415 not (416 or 419 or 422 or 425) [PSYCINFO RECORDS] (40)  429 (2018122* or 2018123* or 2019*).up. (5764288)  430 428 and 429 [PSYCINFO UNIQUE RECORDS - UPDATE PERIOD] (2) | |
| QUALITATIVE SUPPLEMENTAL SEARCH (Sample based on Medline) |  |
| 1 Monitoring, Ambulatory/ (7758)  2 Electrocardiography, Ambulatory/ (10510)  3 Telemedicine/ (19678)  4 Remote Consultation/ (4550)  5 Remote Sensing Technology/ (2475)  6 Telemetry/ (9495)  7 (remote* adj3 (manag* or monitor*)).tw,kf. (3476)  8 ((outpatient* or out-patient* or ambulator* or home? or homebased or home-based) adj3 (manag* or monitor*)).tw,kf. (24054)  9 ((dynamic* or Holter) adj3 (manag* or monitor*)).tw,kf. (11416)  10 ((internet* or app or apps or smarthome* or smarthome* or smartphone* or smart phone* or mobile-based or e-mail* or email* or electronic mail* or emedicine or e-medicine or technolog* or computer* or digital* or webbased or web-based or webdeliver* or web-deliver* or online) adj3 (manag* or monitor*)).tw,kf. (14972)  11 (telemonitor* or tele-monitor*).tw,kf. (1529)  12 (telecare or tele-care or telehealth or tele-health or telemedicine or tele-medicine).tw,kf. (14551)  13 (ehealth or e-health).tw,kf. (5336)  14 (mobile health or mhealth or m-health).tw,kf. (5628)  15 (telemetr* or tele-metr*).tw,kf. (8647)  16 (remote sensing or remote sensor*).tw,kf. (7183)  17 or/1-16 [REMOTE MONITORING] (116758)  18 exp Cardiac Pacing, Artificial/ (23931)  19 (artificial* adj2 card* pac*).tw,kf. (195)  20 (cardiac adj (resynchroni* or re-synchroni*)).tw,kf. (7442)  21 exp Pacemaker, Artificial/ (26329)  22 (pacemak* or pace-mak*).tw,kf. (37350)  23 Defibrillators, Implantable/ (15480)  24 (implant* adj3 defibrillat*).tw,kf. (15005)  25 (implant* adj3 device*).tw,kf. (17430)  26 ((cardiac* or cardio*) adj3 implant*).tw,kf. (17296)  27 (ICD or ICDs).tw,kf. and (exp Heart/ or (cardio* or cardiac* or coronar* or heart? or defibrillat* or fibrillat* or implant*).tw,kf.) (11386)  28 (AICD or AICDs or S-ICD or S-ICDs or CIED or CIEDs).tw,kf. (2374)  29 "Atlas II".tw,kf. (5)  30 (AnalyST Accel or Belos or Lexos or Tachos).tw,kf. (7)  31 (Durata or Dynagen or Energen).tw,kf. (154)  32 Epic DR.tw,kf. (0)  33 "Ilivia 7".tw,kf. (0)  34 Incepta.tw,kf. (2)  35 InSync.tw,kf. (75)  36 (Intica or Inventra or Iperia 7).tw,kf. (0)  37 (Lumax or Lumos).tw,kf. and (exp Heart/ or (cardio* or cardiac* or coronar* or heart? or defibrillat* or fibrillat* or implant*).tw,kf.) (18)  38 ("Maximo II" or "Maximo 2").tw,kf. (0)  39 Micro Jewel.tw,kf. (5)  40 Paradym.tw,kf. (8)  41 Riata Model 7070.tw,kf. (0)  42 Teligen.tw,kf. (6)  43 Ventak.tw,kf. (75)  44 ("remote view" and Medtronic).tw,kf. (0)  45 Electrophysiology/ (79692)  46 Cardiac Electrophysiology/ (408)  47 (electrophysiolog* or electro-physiolog*).tw,kf. (107188)  48 Monitoring, Physiologic/ and (exp Heart/ or (cardio* or cardiac* or coronar* or heart? or defibrillat* or fibrillat* or implant*).tw,kf.) (14235)  49 or/18-48 [ICDs] (266694)  50 17 and 49 [REMOTE MONITORING - ICDs] (7523)  51 interview*.mp. (362119)  52 experience*.mp. (997271)  53 qualitative.tw,kf. (208580)  54 or/51-53 [HIRU - QUALITATIVE FILTER] (1398611)  55 50 and 54 [REMOTE MONITORING - ICDs - HIRU - QUALITATIVE FILTER] (714)  56 Focus Groups/ (27009)  57 exp "Surveys and Questionnaires"/ (961870)  58 focus group?.tw,kf. (41554)  59 survey*.tw,kf. (598502)  60 questionnaire?.tw,kf. (470444)  61 self-report*.tw,kf. (141243)  62 barrier?.tw,kf. (268955)  63 facilitat*.tw,kf. (485713)  64 experiential*.tw,kf. (8234)  65 key informant?.tw,kf. (6727)  66 or/56-65 [ADDITIONAL QUALITATIVE TERMS] (2283789)  67 50 and 66 [REMOTE MONITORING - ICDs - ADDITIONAL QUALITATIVE TERMS] (572)  68 exp Heart Diseases/px [psychology] (14601)  69 Monitoring, Ambulatory/px [psychology] (57)  70 Electrocardiography, Ambulatory/px [psychology] (13)  71 Telemetry/px [psychology] (18)  72 exp Cardiac Pacing, Artificial/px [psychology] (63)  73 exp Pacemaker, Artificial/px [psychology] (82)  74 Defibrillators, Implantable/px [psychology] (435)  75 or/68-74 [PERTINENT MESH AS PER TOPIC + PX (PSYCHOLOGY SUBHEADING)] (15034)  76 50 and 75 [REMOTE MONITORING - ICDs - PERTINENT MESH AS PER TOPIC + PX (PSYCHOLOGY SUBHEADING)] (54)  77 exp Adaptation, Psychological/ (121961)  78 Attitude/ (45763)  79 Attitude to Death/ (15592)  80 exp Attitude to Health/ (392957)  81 ((death? or dying or health*) adj3 attitude?).ti,ab,kf. (8729)  82 Choice Behavior/ (30996)  83 Consumer Advocacy/ (3311)  84 *Consumer Behavior/ (9735)  85 Cooperative Behavior/ (41795)  86 Decision Making/ (89217)  87 Depression/ (109870)  88 depress*.ti,ab,kf. (434413)  89 dignity.ti,ab,kf. (6440)  90 exp Emotional Adjustment/ (761)  91 Emotions/ (62166)  92 emotion*.ti,ab,kf. (182328)  93 Anxiety/ (75275)  94 ((accept* or anxiet* or anxious* or attitud* or consider* or concern* or dissatisf* or expect* or experienc* or fear* or feel* or felt or knowledge* or opinion* or perceiv* or percepti* or perspective? or prefer* or respons* or satisf* or uncertain* or understand* or unsatisf* or value? or valuing or view* or worrie? or worry*) adj3 (female? or male? or men or patient? or person$2 or personally or public or stake?holder* or user? or wom#n or "woman's" or "women's")).ti,ab,kf. (730715)  95 Health Care Surveys/ (30877)  96 Health Services Accessibility/ (69355)  97 Interviews as Topic/ (57885)  98 Life Change Events/ (22204)  99 (life adj3 (event? or experience?)).ti,ab,kf. (28424)  100 Narration/ (7682)  101 Patient Acceptance of Health Care/ (42706)  102 Patient Advocacy/ (23474)  103 exp Patient-Centered Care/ (18316)  104 exp Patient Education as Topic/ (82435)  105 Patient Participation/ (24044)  106 Patient Preference/ (7289)  107 Patient Reported Outcome Measures/ (3389)  108 Patient Satisfaction/ (77452)  109 exp Patients/px (16147)  110 Personal Autonomy/ (16317)  111 ((autonom* or freedom) adj3 (patient? or person$2 or personally or self)).ti,ab,kf. (11235)  112 *"Power (Psychology)"/ (5482)  113 Quality of Life/px (23971)  114 exp Self Concept/ (104419)  115 Self Efficacy/ (18637)  116 (self-concept* or self-efficac* or self-perception*).ti,ab,kf. (34779)  117 (("limit" or limits or limitation* or limited or limiting or change*) adj3 (activit* or work* or employ* or productiv* or autonom* or independen* or freedom or self)).ti,ab,kf. (118720)  118 (cope or coped or copes or coping).ti,ab,kf. (78150)  119 exp Self-Help Groups/ (9921)  120 Social Values/ (19500)  121 Uncertainty/ (11266)  122 ((accept* or anxiet* or anxious* or attitud* or consider* or choice? or choos* or chose? or concern* or decid* or decis* or dissatisf* or expect* or experienc* or fear* or feel* or felt or input* or knowledge* or opinion* or participat* or perceiv* or percepti* or perspective? or prefer* or respons* or satisf* or uncertain* or understand* or unsatisf* or value? or valuing or view* or worrie? or worry*) adj3 (citizen? or client? or consumer? or female? or male? or men or parent$2 or patient? or person$2 or personally or public or stake?holder* or user? or wom#n or "woman's" or "women's")).tw,kf. (875761)  123 (advoca* adj3 (client? or consumer? or patient? or person$2 or personally or wom#n or "woman's" or "women's")).tw,kf. (6124)  124 ((analys#s or valuation? or value? or valuing) adj3 (conjoint or contingent)).tw,kf. (1557)  125 (autonom* adj3 (person$2 or personally or self)).tw,kf. (2662)  126 (choice? adj1 (discrete or experiment*)).tw,kf. (3062)  127 ((client? or consumer? or patient? or person$2 or personally or wom#n or "woman's" or "women's") adj (centered or centred or focus*)).tw,kf. (29989)  128 ((client? or consumer? or patient? or person$2 or personally or wom#n or "woman's" or "women's") adj narrati*).tw,kf. (1381)  129 empower*.tw,kf. (23183)  130 (freedom? or libert*).tw,kf. (46420)  131 gambl*.tw,kf. (8961)  132 ((health or death) adj3 (anxiet* or anxious* or attitud* or concern* or fear* or feel? or feeling* or felt or perception* or perspective? or prefer* or view* or worrie? or worry*)).tw,kf. (75846)  133 health utilit*.tw,kf. (1917)  134 (life adj3 (event? or experience?)).tw,kf. (28424)  135 (multi?attribute or multi?criteria).tw,kf. (1003)  136 (preference? adj1 (elicit* or scor* or stated)).tw,kf. (1610)  137 prospect theor*.tw,kf. (293)  138 ((person$2 or self) adj2 (conceiv* or concept* or percepti* or perceiv*)).tw,kf. (25654)  139 ((person$2 or self) adj2 (determin* or efficac* or help or manag* or support*)).tw,kf. (64941)  140 (social* adj1 valu*).tw,kf. (2013)  141 (trade?off? or tto).tw,kf. (7141)  142 (willing* adj2 pay*).tw,kf. (5744)  143 exp Communication/ (290711)  144 ((time$2 or timeliness) adj2 (communica* or info*)).tw,kf. (7344)  145 (miscommunicat* or mis-communicat*).tw,kf. (799)  146 (misunderstand* or mis-understand*).tw,kf. (5250)  147 (misinform* or mis-inform*).tw,kf. (2675)  148 ((involv* or participat*) adj3 (client? or consumer? or patient? or person$2 or personally or wom#n or "woman's" or "women's")).tw,kf. (110077)  149 exp Informed Consent/ (39852)  150 (informed adj3 (choice* or choos* or consent* or decid* or decision*)).tw,kf. (47514)  151 ((client? or consumer? or patient? or person$2 or personally or wom#n or "woman's" or "women's") adj3 consent*).tw,kf. (14304)  152 (choice? adj2 behavio?r*).tw,kf. (2739)  153 ((guide? or guiding or make or making or makes or made or shar* or support*) adj2 (choice? or choos* or consent* or decid* or decision*)).tw,kf. (176080)  154 or/77-153 [PATIENT PREFERENCES & VALUES] (2863151)  155 50 and 154 [REMOTE MONITORING - ICDs - PATIENT PREFERENCES & VALUES] (1382)  156 exp Professional-Patient Relations/ (138097)  157 ((nurse* or doctor? or personnel or physician? or practitioner? or professional? or provider? or specialist? or cardiologist? or staff or surgeon? or technician? or technologist?) adj3 patient? adj5 relation*).tw,kf. (28777)  158 Attitude of Health Personnel/ (116029)  159 ((accept* or anxiet* or anxious* or attitud* or consider* or choice? or choos* or chose? or concern* or decid* or decis* or dissatisf* or expect* or experienc* or fear* or feel* or felt or input* or knowledge* or opinion* or participat* or perceiv* or percepti* or perspective? or prefer* or respons* or satisf* or uncertain* or understand* or unsatisf* or value? or valuing or view* or worrie? or worry*) adj3 (nurse* or doctor? or personnel or physician? or practitioner? or professional? or provider? or specialist? or cardiologist? or staff or surgeon? or technician? or technologist?)).tw,kf. (222153)  160 ((involv* or participat*) adj3 (nurse* or doctor? or personnel or physician? or practitioner? or professional? or provider? or specialist? or cardiologist? or staff or surgeon? or technician? or technologist?)).tw,kf. (37980)  161 or/156-160 [ADDITIONAL PHYSICIAN/CLINICAL PERSONNEL TERMS] (444352)  162 50 and 161 [REMOTE MONITORING - ICDs - ADDITIONAL PHYSICIAN/CLINICAL PERSONNEL TERMS] (127)  163 55 or 67 or 76 or 155 or 162 [ALL QUALITATIVE/MIXED METHODS SETS] (2154)  164 "Value of Life"/ (5651)  165 Quality of Life/ (177737)  166 quality of life.ti,kf. (76432)  167 ((instrument or instruments) adj3 quality of life).ab. (3206)  168 Quality-Adjusted Life Years/ (11127)  169 quality adjusted life.ti,ab,kf. (11718)  170 (qaly* or qald* or qale* or qtime* or life year or life years).ti,ab,kf. (18668)  171 disability adjusted life.ti,ab,kf. (2949)  172 daly*.ti,ab,kf. (2715)  173 (sf36 or sf 36 or short form 36 or shortform 36 or short form36 or shortform36 or sf thirtysix or sfthirtysix or sfthirty six or sf thirty six or shortform thirtysix or shortform thirty six or short form thirtysix or short form thirty six).ti,ab,kf. (24132)  174 (sf6 or sf 6 or short form 6 or shortform 6 or sf six or sfsix or shortform six or short form six or shortform6 or short form6).ti,ab,kf. (1993)  175 (sf8 or sf 8 or sf eight or sfeight or shortform 8 or shortform 8 or shortform8 or short form8 or shortform eight or short form eight).ti,ab,kf. (432)  176 (sf12 or sf 12 or short form 12 or shortform 12 or short form12 or shortform12 or sf twelve or sftwelve or shortform twelve or short form twelve).ti,ab,kf. (5298)  177 (sf16 or sf 16 or short form 16 or shortform 16 or short form16 or shortform16 or sf sixteen or sfsixteen or shortform sixteen or short form sixteen).ti,ab,kf. (32)  178 (sf20 or sf 20 or short form 20 or shortform 20 or short form20 or shortform20 or sf twenty or sftwenty or shortform twenty or short form twenty).ti,ab,kf. (394)  179 (hql or hqol or h qol or hrqol or hr qol).ti,ab,kf. (15567)  180 (hye or hyes).ti,ab,kf. (66)  181 (health* adj2 year* adj2 equivalent*).ti,ab,kf. (48)  182 (pqol or qls).ti,ab,kf. (379)  183 (quality of wellbeing or quality of well being or index of wellbeing or index of well being or qwb).ti,ab,kf. (547)  184 nottingham health profile*.ti,ab,kf. (1130)  185 sickness impact profile.ti,ab,kf. (1063)  186 exp health status indicators/ (287328)  187 (health adj3 (utilit* or status)).ti,ab,kf. (66859)  188 (utilit* adj3 (valu* or measur* or health or life or estimat* or elicit* or disease or score* or weight)).ti,ab,kf. (11104)  189 (preference* adj3 (valu* or measur* or health or life or estimat* or elicit* or disease or score* or instrument or instruments)).ti,ab,kf. (9863)  190 disutilit*.ti,ab,kf. (428)  191 rosser.ti,ab,kf. (95)  192 willingness to pay.ti,ab,kf. (4957)  193 standard gamble*.ti,ab,kf. (829)  194 (time trade off or time tradeoff).ti,ab,kf. (1361)  195 tto.ti,ab,kf. (992)  196 (hui or hui1 or hui2 or hui3).ti,ab,kf. (1390)  197 (eq or euroqol or euro qol or eq5d or eq 5d or euroqual or euro qual).ti,ab,kf. (14228)  198 duke health profile.ti,ab,kf. (84)  199 functional status questionnaire.ti,ab,kf. (123)  200 dartmouth coop functional health assessment*.ti,ab,kf. (12)  201 (WHOQOL or WHOQOL-BREF).ti,ab,kf. (2580)  202 "cost of illness"/ (25278)  203 (cost? adj3 illness*).ti,ab,kf. (2787)  204 exp Disability Evaluation/ (50124)  205 ((disabil* or disabled or impaired or impairment*) adj3 (estimat* or evaluat* or instrument or instruments or measur* or scale? or score? or weight? or valu*)).ti,ab,kf. (36711)  206 burden*.ti,ab,kf. (192636)  207 (toll or tolls).ti,ab,kf. (40857)  208 exp Severity of Illness Index/ (236292)  209 ((disease* or illness* or sickness*) adj3 sever* adj2 (estimat* or evaluat* or instrument or instruments or measur* or scale? or score? or weight? or valu*)).ti,ab,kf. (7013)  210 ((disease* or illness* or sickness*) adj2 impact?).ti,ab,kf. (8140)  211 Absenteeism/ (8749)  212 absentee*.ti,ab,kf. (5747)  213 Presenteeism/ (217)  214 presentee*.ti,ab,kf. (1188)  215 productivit*.ti,ab,kf. (54103)  216 ((work* or employ*) adj5 (absenc* or absent* or presenc* or present*)).ti,ab,kf. (112562)  217 ((work* or employ*) adj5 abilit*).ti,ab,kf. (10863)  218 (time adj1 away).ti,ab,kf. (609)  219 Sick Leave/ (5493)  220 ((sick or medical) adj leave).ti,ab,kf. (5026)  221 or/164-220 [QoL/Burden] (1003098)  222 50 and 221 [ICDs - REMOTE MONITORING - QoL/BURDEN] (529)  223 163 or 222 [ALL QUALITATIVE/MIXED METHODS SETS, INCL QoL/BURDEN] (2367)  224 exp Animals/ not Humans/ (4592769)  225 223 not 224 [ANIMAL-ONLY REMOVED] (2250)  226 (news or newspaper article).pt. (212417)  227 225 not 226 [NEWS ITEMS REMOVED] (2249)  228 limit 227 to yr="2000-current" (1517)  229 2019*.dt. (642679)  230 228 and 229 (50) | |
